# Thematic analysis of how general practitioners perceive digital social prescribing as an intervention aiming at promoting psychosocial health and wellbeing in older adults

**DOI:** 10.3389/fpubh.2026.1754026

**Published:** 2026-03-25

**Authors:** Rashid Menhas, Nasiru Mohammad Abdullahi, Patience Osose Nasir, Mohamed Goda Elbqry, Fatma Mohamed Elmansy, Saddam Ahmed Al-Ahdal, Ashwaq Hamad Alshmimry, Azza Elsayed Abd elfatah Arafat, Samia Eaid Elgazzar, Fatima S. O. Ashmieg

**Affiliations:** 1 School of Nursing, Shandong Xiehe University, Jinan, China; 2 Department of Community, Psychiatric and Mental Health Nursing, College of Nursing, Qassim University, Buraydah, Saudi Arabia; 3 Department of Medical-Surgical Nursing, College of Nursing Sciences, Inaya Medical Colleges, Riyadh, Saudi Arabia; 4 Department of Medical-Surgical Nursing, College of Nursing, Qassim University, Buraydah, Saudi Arabia; 5 Department of Nursing, Medical City, Qassim University, Buraydah, Saudi Arabia

**Keywords:** DSP, GP, health and wellbeing, older adults, psycho-social

## Abstract

**Background:**

Population aging has intensified the need for interventions that address the psychosocial health and wellbeing of older adults. Digital social prescribing (DSP), an extension of traditional social prescribing, uses digital technologies to connect individuals with non-medical community resources and may help address social isolation and loneliness in older populations.

**Objective:**

This study explored general practitioners’ (GPs) perceptions of digital social prescribing as an intervention to promote psychosocial health and wellbeing among older adults in China.

**Methods:**

A qualitative exploratory design was employed in Yiwu, Zhejiang Province, China. Semi-structured interviews were conducted with 27 general practitioners working in community health centers and clinics. Participants were recruited using convenience sampling methods. The interview data were analyzed using reflexive thematic analysis.

**Results:**

Six key themes emerged: (i) digital technology adoption, (ii) social support networks, (iii) healthcare system integration, (iv) implementation challenges, (v) cultural and contextual adaptation, and (vi) policy and practice recommendations. GPs generally perceived DSP as a feasible and valuable approach to addressing psychosocial issues such as social isolation, loneliness, and poor mental health among older adults. However, concerns have been raised regarding digital literacy, workload, infrastructure, and data security.

**Conclusion:**

General practitioners expressed positive attitudes toward the use of digital social prescribing to support the psychosocial wellbeing of older adults. With appropriate training, policy support, and culturally adapted implementation, DSP has the potential to address the key social determinants of health and complement existing primary care services in rapidly aging societies.

## Introduction

According to a World Health Organization (WHO) report, the population of older adults is expected to increase from 12% in 2015 to 22% in 2050, with the largest increase in low- and middle-income countries (LMICs) (1). This trend is prominent in the Western Pacific Region, where China will have 402 million citizens aged 60 years or older by 2040, comprising 28% of its population (2). In China, those aged ≥65 years now exceed 216.76 million, representing 15.4% of the population based on the latest estimates. Predictions indicate more than 300 million cases by 2035 (3). This is due to reduced fertility, improved life expectancy, and the effects of the “one-child” policy and family planning programs (4).

The psychosocial health of older adults is an urgent problem, as aging increases the risk of mental health disorders, social isolation, loneliness, depression and anxiety. Research shows that 27% of community-living older adults in China are affected by frailty (5). Urbanization and a lack of community facilities have worsened this situation. CHARLS found that 30% of older people in China felt depressed, leading to nutritional imbalance and low quality of life due to a lack of communication (6). This issue damages individuals’ health and burdens medical systems through increased hospitalizations and healthcare costs (7). The pandemic has worsened this problem, with mandatory distancing causing a 28.1% increase in psychological distress worldwide (8).

Social prescribing (SP) addresses the limitations of traditional medical interventions, such as drug therapy or counseling, which overlook other health-related factors. It involves healthcare staff referring patients to community resources and activities to help address loneliness, financial pressure, and a lack of purpose (9). SP promotes wellness, decreases hospital visits, and increases life satisfaction in patients. In the UK’s NHS, surveys show 63% of GPs find it useful for non-health-related issues (10). Digital social prescribing (DSP), a digital version of social prescribing, is a promising development that may benefit the geriatric population, particularly those with mobility or distance-related barriers. Digital social prescribing (DSP) includes smartphone applications, web-based platforms, VR, and telematics, through which older adults can access nonclinical services and support, such as remote group activities, digital skills training, and online hobbies. This aligns with the digital health revolution (11). mHealth apps have empowered users and promoted intervention adherence (12). DSP offers convenience to those living in remote areas. In rural South Korea, participants experienced less depression or anxiety after connecting with nearby community resource centers through an application (13). However, the adoption of digital social prescribing (DSP) is slow because of low digital literacy (14).

**Figure 1:**
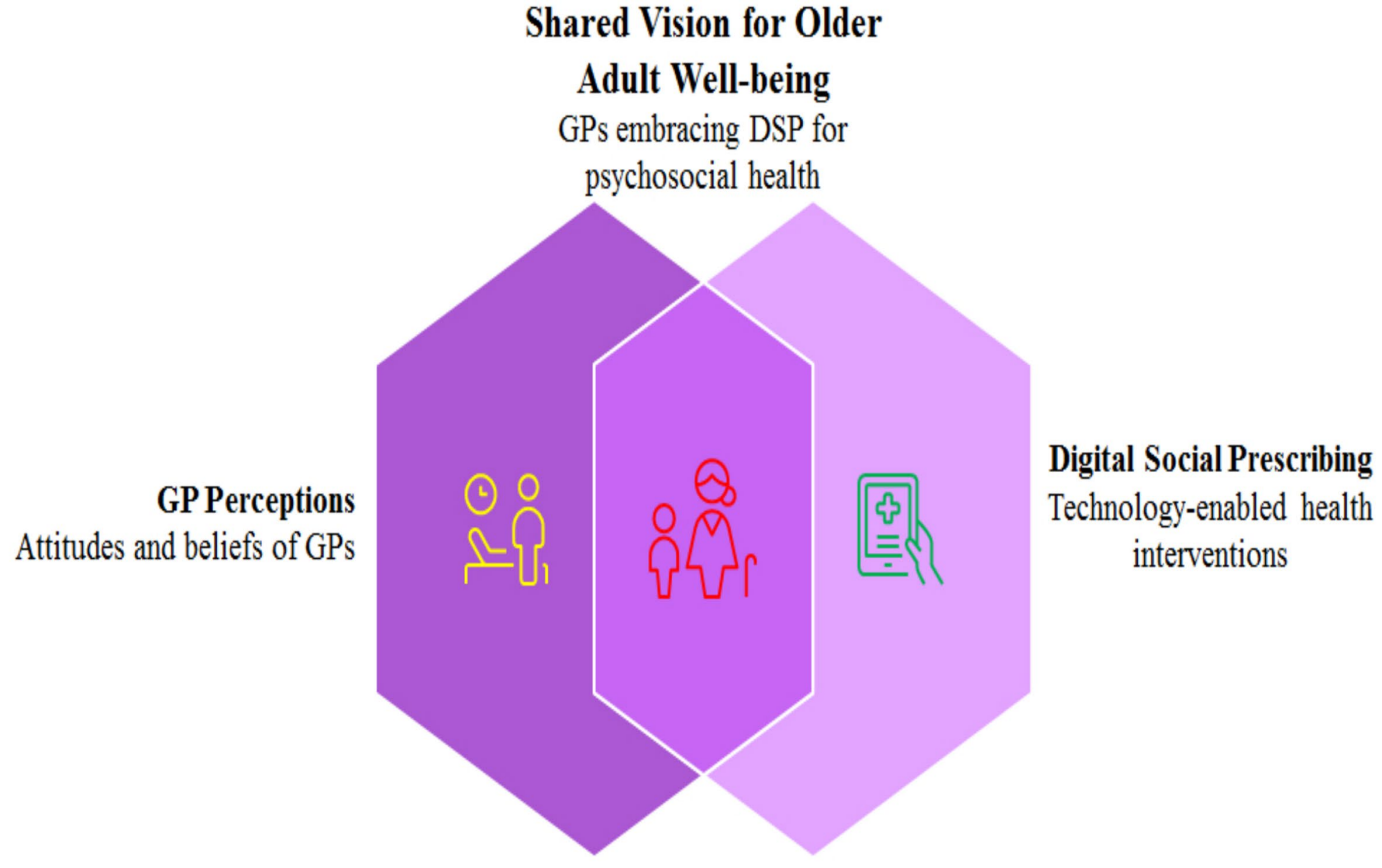
Conceptual framework.

China has over 1 billion Internet users, with a digital divide among older adults (15). Research has shown that 25% of older adult patients at community health centers have psychological and social problems, requiring new interventions (16). Surveys in Singapore and Japan have indicated that while medical staff supported digital solutions, digital disparity remained a barrier (17, 18). Aging increases vulnerability to psychosocial issues, impacting individuals and healthcare systems, particularly during urbanization and pandemics. Social prescribing (SP) addresses traditional medical limitations by connecting patients with community resources. Digital social prescribing (DSP) extends SP through technology to overcome mobility barriers, although digital literacy gaps limit its adoption. China’s digital growth and aging population make it ideal for studying DSP, with Yiwu facing significant challenges in the provision of geriatric care. GPs are crucial for recommending digital social prescribing (DSP). However, few studies (13, 17, 18) have examined these perspectives in non-Western contexts. This study analyzed GPs’ perceptions of digital social prescribing (DSP) as an intervention for promoting psychosocial health in older adults (See Figure 1).

### Theoretical framework

**Figure 2:**
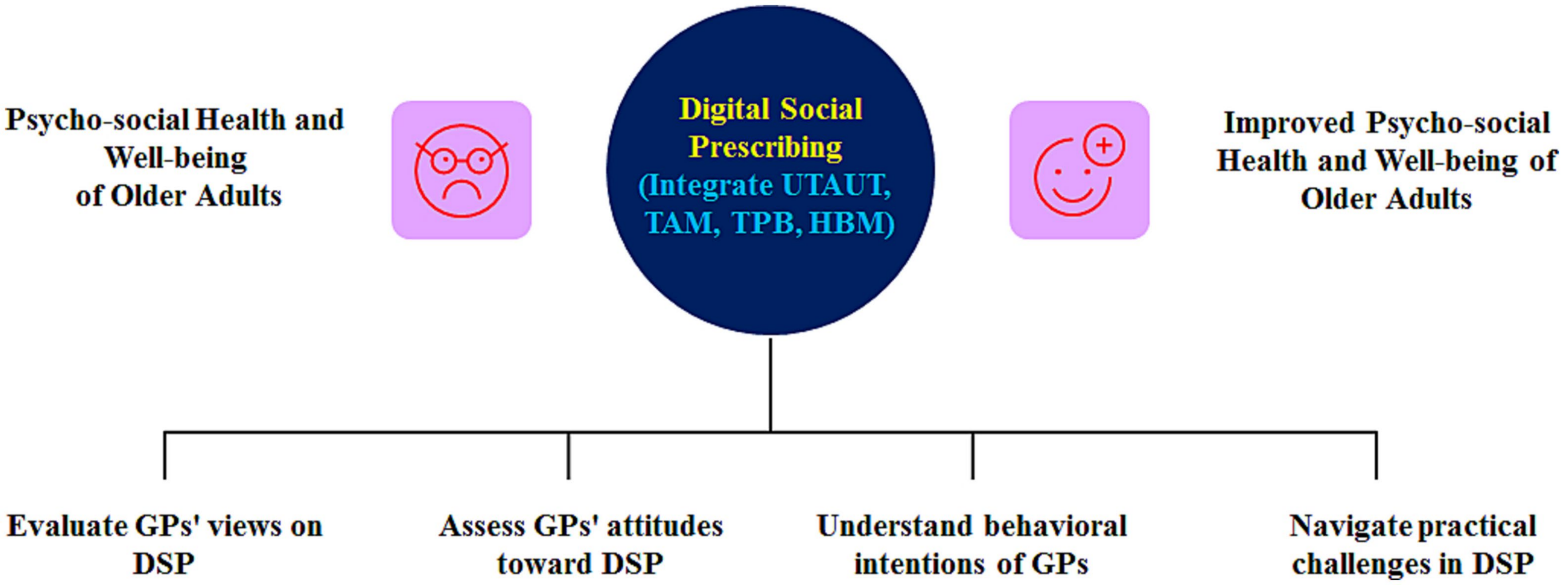
Theoretical framework.

The technology acceptance model (TAM) and theory of planned behavior (TPB) explain users’ intentions through four key variables: performance expectancy, effort expectancy, social influence, and facilitating conditions. From the unified theory of acceptance and use of technology (UTAUT) perspective, this study explored GPs’ views on digital social prescribing (DSPs), their perceived advantages, and obstacles to better serve older people’s psychological and social problems. Performance expectancy refers to GPs’ belief that digital social prescribing (DSP) provides greater value than paper-based prescribing (19). GPs believe that digital social prescribing (DSPs) help them find local online groups/courses, helping older people reduce loneliness and helplessness, especially when they are unable to go out. We draw on TAM ideas regarding perceived usefulness and perceived ease of use as separate dimensions. To assess GPs’ attitudes toward digital social prescribing (DSPs), effort expectancy focuses on ‘digital access’ and ‘workload impact’ barriers: GPs worry that learning to use unfamiliar systems will be too difficult. Based on UTAUT theory, many positive results have emerged regarding digital technology in medical care. One mHealth acceptance study showed that performance and effort expectancy predicted healthcare workers’ behavioral intention, while social influence strengthened information-sharing intention (20). Peer and superior word-of-mouth effects are necessary to promote digital social prescribing (DSP) use among the older adults. Older doctors may lack sufficient experience with digital devices, thereby reducing their operating confidence (21). We added the “Health Belief Model” (HBM), specifically “perceived barrier” and “cue to action,” to understand how GPs solve practical problems during use (See Figure 2). The HBM focuses on health context aspects, such as attitudes toward digital technology for psycho-social health and wellbeing (22, 23).

### Statement of the study

The psychosocial wellbeing of older adults is a major public health issue. The proportion of people aged ≥65 years will almost double across countries by 2050 (2). Loneliness, social isolation, and related mental problems (such as depression and anxiety) are common among older adults, leading to higher healthcare demands and poorer quality of life (2). Under the current biomedical orientation in primary care, physical health problems are prioritized over psychosocial problems (8). “Social prescribing” refers to community resources such as groups, clubs, or classes and has been recognized as a promising intervention for addressing loneliness, reducing social isolation, fostering connections, and enhancing emotional resilience (8, 9). However, there is no unified standard for social prescribing across locations, especially for those aged over 65 years living in remote areas, poor environments, and experiencing difficulty in traveling (9). “Digital social prescribing (DSP),” a development form based on new digital technology (such as online platforms, mobile apps, and virtual communities), is worth exploring for older adults to participate in psychosocial health interventions remotely (11). Chatbots and voice assistants, as types of conversational artificial intelligence (CAI), can provide company, daily aids, and health advice, thereby satisfying social care needs (24). Digital social prescribing (DSP) presents opportunities to increase service access, prevent mental health deterioration, enhance the cost-effectiveness of mental health care, and complement existing social prescribing referrals with timely recommendations (11, 12). In primary healthcare, general practitioners (GPs) typically make the initial referral decisions for older adults. Most GPs view social prescribing favorably as a means to address psychosocial rather than physical problems, such as tackling loneliness and promoting emotional wellbeing (25). No previous study has synthesized evidence related to digital social prescribing and the psychological and social needs of older people from GPs’ perspective of GPs. For implementation science concerning mental health among older adults in the Western Pacific region, more evidence is needed on scaling up social prescribing while recognizing uncertainty about what works in practice and considering the context (26). Although barrier and promoter analyses have been conducted for older people’s participation in social prescribing, the incorporation of digital social prescribing (DSP) has not been studied qualitatively by GPs (27). Such gaps prevent us from developing interventions/policies based on needs because none of them considered how they would be accepted by GPs in their daily clinical work. Following research questions were designed to achieve the study objective.

*RQ1:* How do GPs define and conceptualize digital social prescribing (DSP) within the geriatric psychosocial health context for older adults?

*RQ2:* What do GPs think about the perceived benefits of digital social prescribing (DSP)?

*RQ3:* What barriers are there related to digital social prescribing (DSP) from the perspective of GPs, and how can they be solved?

## Methods

### Study locale

This study was conducted in Yiwu, Zhejiang Province, China. Yiwu faces an increasingly “aged” population due to migration (14). In the context of an increasing proportion of older people, this trend is expected to intensify amid the demographic changes in China. Older adults struggle with loneliness and other psychological issues. Ethical approval (K2023034) for this study was granted by the Fourth Affiliated Hospital of Zhejiang University School of Medicine, Yiwu, Zhejiang, China. This study adhered to the principles of the Declaration of Helsinki.

### Study design

A qualitative exploratory research design was employed. Semi-structured interviews were conducted with GPs to understand how they define and perceive digital social prescribing (DSP) as a means of promoting the psychosocial health of older people. No pre-established hypotheses were used in the study design. The topic of psychosocial health and wellbeing in older age is highly dependent on both individual experience and structural context (28). The thematic analysis approach was chosen as the basis for analyzing the primary data.

### Study participants

Convenience sampling was used to invite 27 general practitioners (GPs) to participate in the study. In this study, we used convenience sampling because of its compatibility with our exploratory research goals and the practical limitations of reaching the intended population. This non-probability sampling method involves choosing participants who are easily accessible to the researchers rather than randomly selecting them from a specified population frame. This method was considered suitable for our research as our aim was to gain initial insights into general practitioners’ views on digital social prescribing as a means to enhance psychosocial health and wellbeing in older adults, rather than to achieve statistical generalization to a larger population. In exploratory studies such as ours, convenience sampling allows for efficient data collection while providing rich qualitative insights from participants willing to engage. All invited general practitioners worked at community health centers and public clinics in the city. To be included in our research, they had to hold a valid license and work as a GP in Yiwu City for >1 year. In addition, their current workload must include the treatment of older adults (aged ≥ 65 years) as regular patients.

### Development and validation of a data collection tool

The development of the data collection tool (i.e., interview guide) was based on our literature review and theoretical framework to identify demographic characteristics, definitions of digital social prescribing (DSP), awareness, attitudes toward digital social prescribing (DSP), enablers and barriers, strategies to address problems, integration, and suggestions. Thus, we divided the interview guide into two sections: demographics and main questions, aligned with the study objective. After developing the preliminary guides, we consulted three experts in geriatrics and two researchers specializing in qualitative research to ensure that the guides were clear, appropriate, and not offensive to any ethnic group or community in our region. The model was then validated in a stepwise manner. First, content validity was assessed by five experts using the content validity index (CVI) method, yielding a good coefficient of 0.92 (>0.8 acceptable) (29). Second, we conducted pretests with three local general practitioners outside our study sample in Yiwu City via simulated interviews lasting approximately 45–60 min. Based on their opinions and comments, some items required further explanation, such as what exactly “digital social prescribing” means. Additionally, we rearranged all topics to prevent participants from becoming tired after prolonged listening (See Supplementary File).

### Data collection

Primary data were collected through semi-structured interviews conducted between March and July of 2024. After explaining the study objectives, written informed consent was obtained from the individual(s) for the publication of any potentially identifiable images or data included in this article. We provided each interviewee with a participant information form that clearly stated the study’s purpose. The investigators ensured that the participants’ personal privacy was strictly protected and not disclosed. We arranged the interview locations based on the interviewees’ actual situations. Two Chinese-speaking research assistants conducted all interviews. The interview duration ranged from 45 to 75 min, with an average duration of approximately 58 min. Interviews continued until data saturation was achieved, as evidenced by the emergence of no new themes or insights in the final interview. Simultaneously, capturing the information conveyed through nonverbal behavior is important. They must take detailed field notes during the interview process. Finally, to mitigate the risk of bias caused by the interviewer’s subjective judgment influencing the research results, we implemented two measures: a reflective diary and member check (30).

### Data analysis

**Figure 3:**
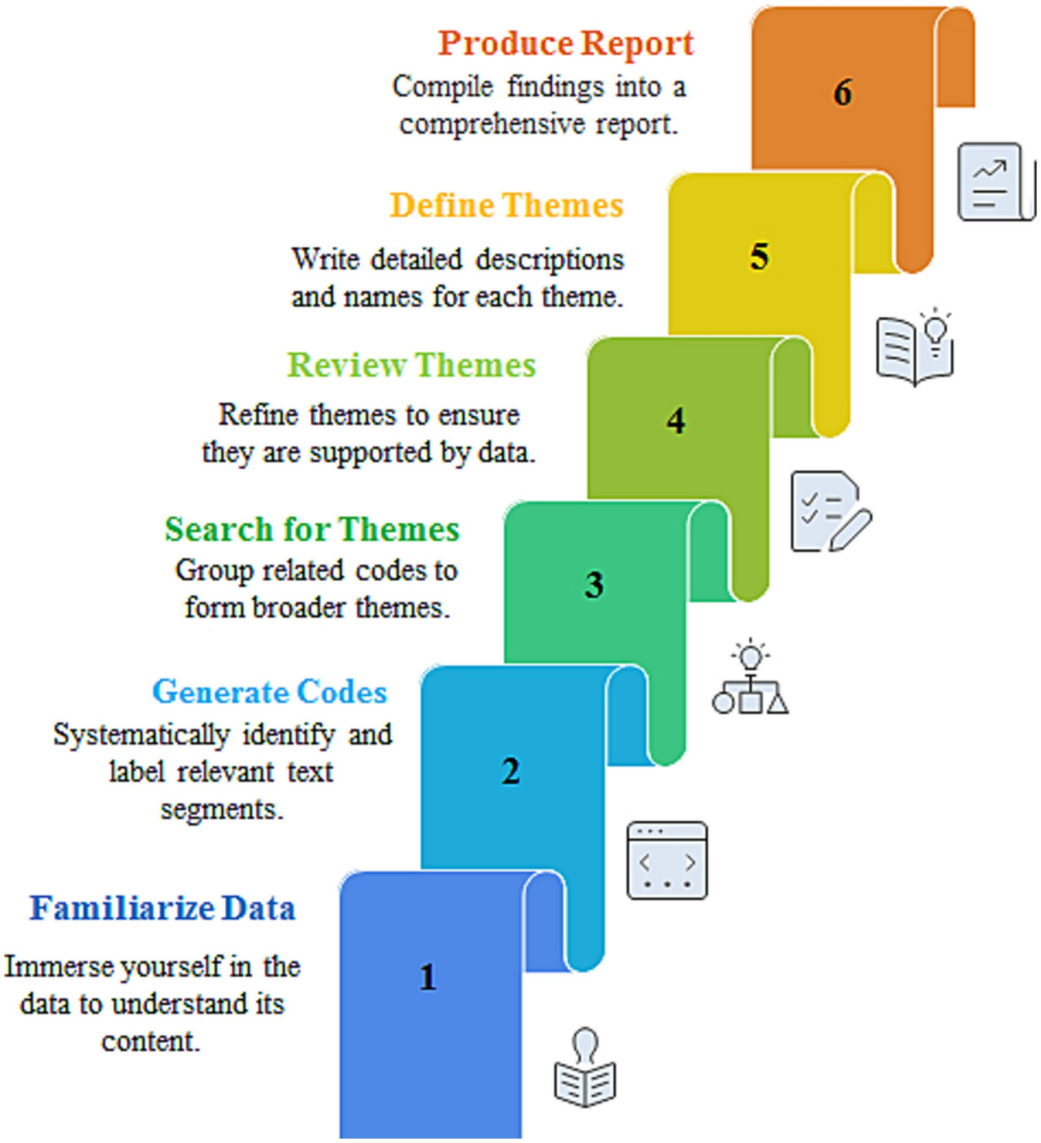
Thematic analysis framework.

Braun and Clarke’s reflexive thematic analysis approach was used to analyze the primary data (31, 32). A six-phase thematic analysis approach is well-suited to achieve the study objectives (See Figure 3). A team of (RM, PON, MGE, FME, SAA, AHA, AEAA, SEE, FSA) researchers performed the thematic analysis.

## Results

### Demographics of the participants

Table 1 depicts the characteristics of the participants (*N* = 27), who are presumably health workers given their focus on the practice setting and care of older patients. The majority of participants were male (66.7%), followed by female (25.9%), and those who preferred not to disclose their gender (7.4%). The age distribution indicates a predominance of younger and middle-aged respondents, with five individuals (18.5%) under 30 years of age, seven (25.9%) aged 30–39, another seven (25.9%) aged 40–49, three (11.1%) aged 50–59, and five (18.5%) aged 60 or above. The years of practice were similarly distributed among both novice and experienced practitioners, with the majority having worked for a shorter duration: “less than 5” years (33.3%), “5–10” years (22.2%), “11–15” years (7.4%), “16–20” years (14.8%), and “>20” years (22.2%). A significant proportion of participants were employed in urban clinics (37.0%), followed by hospital settings (*n* = 8; 29.6%), rural clinics (25.9%), and community health centers (7.4%), highlighting the diversity of their work environment. Finally, the percentage of participants who provided care to older individuals varied considerably: a minority reported <25% (7.4%), some indicated 25–49% (37%), a few reported 50–74% (18.5%), and the remainder reported ≥75% (37%) of their working hours.

**Table 1:** Demographics of the participants (*N* = 27).

Characteristic	Category	*N*	Percentage (%)
Gender	Male	18	66.7%
	Female	7	25.9%
	Prefer not to answer	2	7.4%
Age group	Under 30	5	18.5%
	30–39	7	25.9%
	40–49	7	25.9%
	50–59	3	11.1%
	60 or older	5	18.5%
Practice years	Less than 5 years	9	33.3%
	5–10 years	6	22.2%
	11–15 years	2	7.4%
	16–20 years	4	14.8%
	More than 20 years	6	22.2%
Practice setting	Urban clinic	10	37.0%
	Hospital-based	8	29.6%
	Rural clinic	7	25.9%
	Community health center	2	7.4%
Geriatric patients	Less than 25%	2	7.4%
	25–49%	10	37.0%
	50–74%	5	18.5%
	75% or more	10	37.0%

### Sub-themes analysis

Table 2 presents the subthemes derived from the six main themes identified through the qualitative analysis of interviews with 27 participants. These interviews focused on the integration of digital technologies in digital social prescribing for gerontological care and the frequency of relevant code occurrences in the dataset. Braun and Clarke (31) emphasized that determining what constitutes a theme involves assessing the prevalence of the extent to which a pattern appears in the data. The prevalence scores presented in the thematic analysis table indicate the degree to which each subtheme emerged across various data sources such as participant interviews and focus groups. Higher values indicate greater pervasiveness and relevance within the study sample. For example, subthemes related to Digital Technology Adoption exhibited the highest prevalence, with scores ranging from 17 to 24. Notably, Digital Literacy achieved a peak score of 24, underscoring its widespread occurrence and critical role in challenges related to technology integration. In contrast, Social Support Networks demonstrated moderately high scores of 16–21, highlighting the consistent presence of community and peer dynamics. Meanwhile, the Healthcare System Integration scores ranged from 14 to 18, suggesting balanced but slightly less ubiquitous integration. Implementation Challenges displayed lower scores of 9–13, indicating more localized barriers, while Policy and Contextual Adaptation scores of 12–15 emphasized adaptive strategies as recurrent yet not dominant themes.

**Table 2:** Sub-themes analysis.

Main theme	Sub-theme	Code frequency	Supporting quotes count	Prevalence score
Digital technology adoption	Technology Infrastructure	89	45	22
Digital technology adoption	User Interface Design	76	38	19
Digital technology adoption	Digital Literacy	95	52	24
Digital technology adoption	Platform Integration	67	32	17
Social support networks	Community Connections	78	41	20
Social support networks	Peer Support Networks	82	44	21
Social support networks	Social Activities	65	28	16
Healthcare system integration	Clinical Workflow	71	35	18
Healthcare system integration	Care Coordination	69	33	17
Healthcare system integration	Provider Roles	58	25	14
Implementation challenges	Digital Barriers	45	18	11
Implementation challenges	System Barriers	38	15	9
Cultural and contextual adaptation	Cultural Relevance	52	22	13
Cultural and contextual adaptation	Language Adaptation	48	20	12
Policy and practice recommendations	Training Programs	61	27	15
Policy and practice recommendations	Policy Framework	58	24	14

### Main themes analysis

Table 3 presents the main themes generated from the qualitative dataset. In total, six themes captured the key recurring patterns among participant responses: “Digital technology adoption” (the highest prevalent score is 95, i.e., “digital,” “platform,” “tool/app,” “technology,” “online,” “interface/design,” “simplified,” “voice,” and “access/literacy”). “Social support network” (“social,” “support,” “community/connection,” “activity/group,” “network/isolation,” “family,” and “peer”). “Healthcare system integration” (“integrate/integration,” “care,” “healthcare,” “patient,” “provider,” “clinical,” “treat,” “refer,” “coordinate,” and “workflow”). “Implementation barrier/challenge” (lowest prevalence score is 35, i.e., “barrier/challenge/difficulty,” “limitation/obstacle,” “train,” “confident,” “cost,” “privacy/security”). “Culture/context adaptation” (“culture,” “appropriate,” “China/Yiwu,” “local/community,” “language,” “familiar/traditional,” and “context”). “Politics/practice advice/recommendation” (“policy,” “change,” “recommend,” “reimburse,” “train,” “program,” “standard/guideline,” and “frame/work”).

**Table 3:** Main themes analysis.

Main theme	Key words	Prevalence score
Digital technology adoption	The design of digital platform tools/app technology interfaces will continue to simplify how we interact by voice, how it gives us access, and even teaches our technological literacy.	95
Social support networks	Social Support, Community Connection/Connections, Activity Group/Groups, Social Network/Networks, Social Isolation, Family/Families, Peer/Peers	88
Healthcare system integration	Integrated Care: How can we better integrate health care around patients instead of providers?	72
Implementation challenges	Barriers/challenges/difficulties and limitations/obstacles, training, confidence, costs, privacy and security	35
Cultural and contextual adaptation	In the context of culture, it is suitable for the Chinese to be familiar with the local community language in Yiwu and understand the background of traditional cultures.	42
Policy and practice recommendations	Policy, changes, recommendations, reimbursement, training, programs, standards, guidelines, framework	58

### Finalized main extracted themes

**Figure 4:**
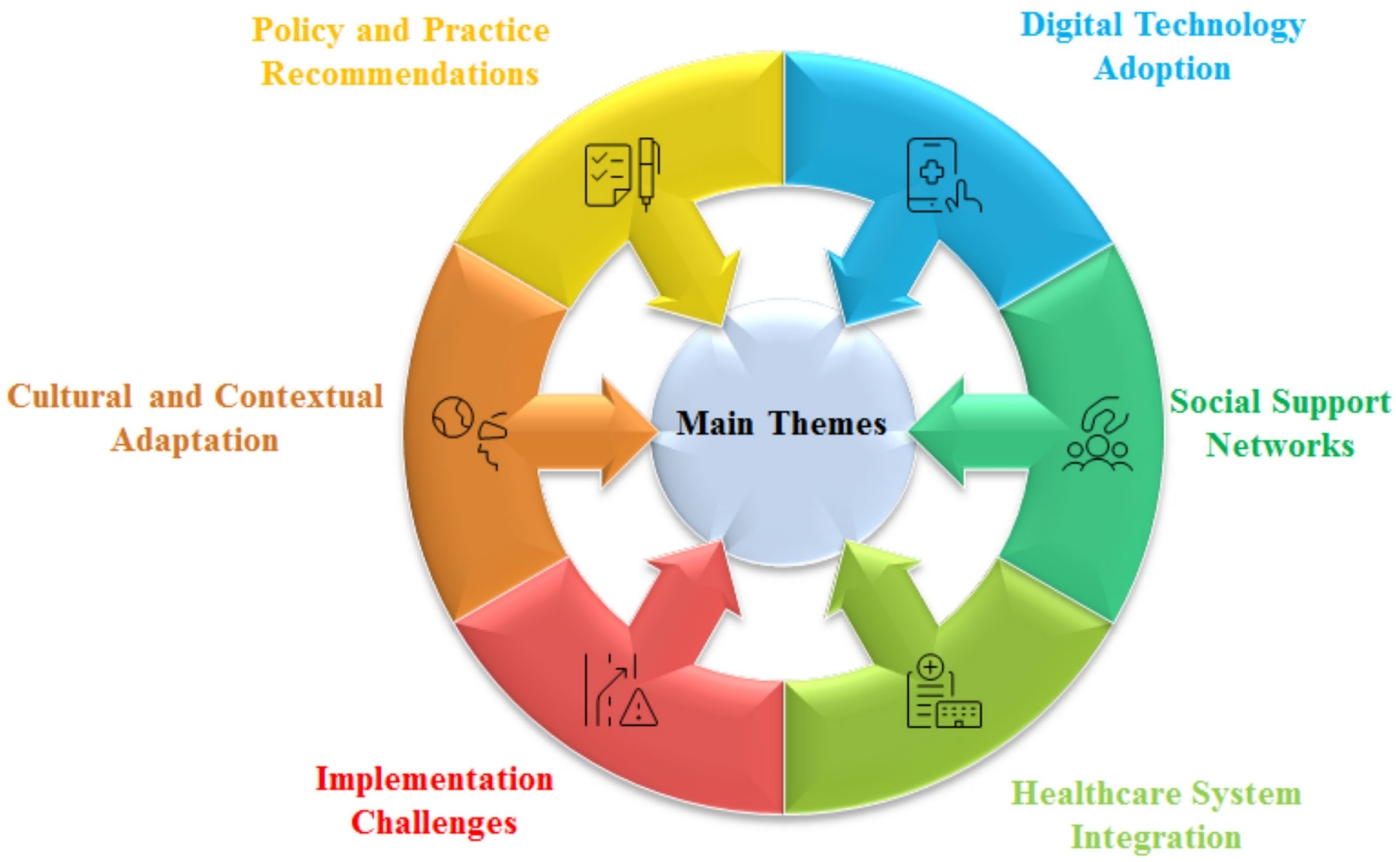
Main extracted themes.

Figure 4 shows main extracted themes.

### Digital technology adoption

The theme “Digital Technology Adoption,” is defined as utilizing technology (i.e., apps/platforms) as tools to deliver social prescribing (SP) for older people, which includes the ease of use of these technologies (See Table 3 and Figure 4). Specifically, this refers to the design of digital interfaces as a means of connecting with social connections/support based on their characteristics, which are specifically designed for older adults (e.g., simple navigation and voice commands). All participants had some level of experience using different kinds of digital devices and recognized the benefits they bring in improving health. However, it should not be forgotten that they must be “easy enough” to encourage them to learn new things.

*Digital social prescribing (DSP) was operationalized by participants through apps, Internet platforms, and interfaces to connect with older patients, focusing on technology literacy training and making it accessible to overcome first-time fear (GP01, GP06, GP26): “So, digital social prescribing is basically digitally connecting patients who are older adults with local services and communities through these different tech platforms and tools available online/app-based. Because otherwise, if they have trouble with moving around physically, then accessing community supports and resources outside their home will become very difficult.” For platform design and development, participants emphasized a simple interface design and integration with familiar applications. “Develop the culture-specific platform applications based on the needs of the older adults in China: simple operational interface, voice operation function; integration with familiar application software such as WeChat or Alipay” (GP01, GP07, GP26)*.

### Social support networks

Social support networks are about building connections online to reduce isolation for older people, including community events, family participation, and peer interaction based on technology (See Table 3 and Figure 4), which stresses the construction of online groups and social relationships to provide emotional and practical help for improving general health and reducing the pressure on medical services.

*Social prescribing (SP) involves establishing support networks by referring older adults to virtual communities, fitness classes, and family connections to promote participation and reduce social isolation. It is like a new way of tackling social determinants of health digitally, referring older adults to virtual support groups, exercise classes, or community events via smartphones or tablets (GP02, GP22). The convenience of these networks was noted (“but one advantage of digital tools is you could provide 24-7 access to supportive networks and resources that’s just not feasible by more conventional means” (GP01, GP06). Integration with existing apps was viewed as important: “a platform which has incorporated local community and WeChat-like interface is more popular amongst our older patients in Yiwu,” showing how culturally compatible networks build social connections and promote psychological health. (GP03; GP23)*.

### Healthcare system integration

To effectively integrate digital social prescribing (DSP) into routine clinical workflows, such as screening and care plan development, it is essential to establish a connection with the existing system to ensure the provision of continuous and comprehensive services (See Table 3 and Figure 4). The formulation of a standardized system, along with IT support, is necessary to enable the scheduling of social interventions in a manner similar to pharmaceuticals or other medical treatments, thereby enhancing operational efficiency and extending the benefits to a larger population.

*Integration of healthcare systems was described as building into existing infrastructure (i.e., putting technology into “the care pathway” (GP07), changing how things normally run (GP06), and creating new jobs). Participants imagined that it would require systematic change, day-to-day screening for social needs at every visit, having approved apps/websites/tools available, and including questions about technology use as part of normal check-ups. (GP01, GP06, GP07). In terms of delivering care in practice, a stepped-care approach was suggested whereby “we start with easy things first and go from there according to how much the person benefits,” which would include care coordinators supporting with set-up and regular check-ins after that (GP02; GP22). It was proposed that an overall approach be taken and social prescribing be integrated into the service model: ‘If we are going to use the integrated approach, then it needs to be delivered within comprehensive care management, with social prescribing being another intervention available to patients as part of their treatment plan, supported by someone trained for this role’ (GP05, GP25).*

### Implementation challenges

Addressing the challenges associated with the implementation of digital social prescribing (DSP) for older adults is imperative. These challenges encompass a range of issues that are encountered during deployment. For instance, what specific technical difficulties are present in the technology (See Table 3 and Figure 4)? Are adequate financial resources available to procure equipment and compensate staff? Have frontline practitioners received appropriate training, and do they perceive the process positively? Furthermore, is the collection of patient information considered intrusive? The inability to resolve these issues could impede the integration and utilization of AI in clinical practices. Consequently, addressing these operational challenges has emerged as a critical concern that warrants focused research.

*Implementation challenges are those related to how realistic it is in practice (i.e., digital divide/literacy issues, cost[s], and security). As one interviewee put it: ‘The key barriers were around digital literacy amongst older people, worry about sharing sensitive information, and that initially, it would take longer for GPs to use such technology’ (GP04, GP24). Other issues were identified relating to resources, such as cost barriers ‘, device Internet fees, technical problem subsidies, and a good support system (GP05; GP25). Some participants highlighted the importance of building confidence: “there are training gaps which cause low confidence amongst providers; therefore, we need to have hands-on training, continued support to overcome such barriers in order not to disrupt service delivery, and increase the feasibility of using technology at the workplace” (GP02, GP07, GP22)*.

### Cultural and contextual adaptation

Cultural context adaptation refers to modifying the design and content of digital social prescribing (DSP) interventions according to the local culture, language preference, and environmental characteristics of older adults in Yiwu (See Table 3 and Figure 4), which is mainly reflected in two aspects: (1) how to inherit and carry forward the local traditional culture and popular activities based on new technology products to increase their sense of identity and recognition and (2) how to pay attention to the differences in regional medical environment and health concept, avoid cultural impact during intervention implementation and promotion, and make them feel respected, trusted, and accepted. All participants agreed that it is essential to consider the factor of locality to gain trust; otherwise, they would not accept it if we simply copied other successful cases.

*Cultural and context-based modifications refer to modifying the platform based on language localization, customs, and community characteristics so that older people can better accept and use it effectively. The participants felt this was necessary because “the design should consider culture, add elements of traditional culture, such as Tai Chi, family activities, so that it will be closer to the hearts of the older people in Yiwu” (GP03; GP23). The importance of familiar tools was highlighted: “In adapting to the local context, use interfaces in Mandarin, integrate with apps such as WeChat that are already part of everyday lives to lower the entry barrier and increase adoption.” (GP01, GP06, GP26) Interviewees proposed taking measures for community alignment because “for successful adaptation, we must cooperate with local communities and include their original social components so that they will feel that new technologies are part of their own culture instead of something imposed from outside” (GP02, GP22)*.

### Policy and practice recommendations

Recommendations on policy and practice are strategies to solve the problem of how to tackle the barrier by system change, such as training, reimbursement policy, assessment method, and so forth, in order to sustain its operation (See Table 3 and Figure 4), which calls on the joint work of all parties to unify standards and stimulate enthusiasm for digital social prescribing.

*To facilitate broader implementation, policies and strategies should prioritize three fundamental components: structural reform, education and training, and outcome evaluation. Specific measures include providing comprehensive training courses for general practitioners (GPs) and patients. Additionally, structured induction training, ongoing technical support, and peer coaching groups are essential for enhancing the security and competence of GPs (GP01, GPO6). It is recommended that policies be amended to favor the reimbursement of services related to digital social prescribing, thereby incentivizing GP participation. Furthermore, standardized evaluation measures are necessary (GP03, GP04, GP23 and GP24). The importance of evaluation is emphasized: an evaluation framework should be established with clearly defined outcome measures, including a loneliness scale, Quality of Life (QoL) index, and health service utilization metrics, to demonstrate the benefits (GP03, G023)*.

## Discussion

The present study offers valuable insights into general practitioners’ (GPs) perspectives on digital social prescribing (DSP) as a means of enhancing the psychosocial health and wellbeing of older adults in Yiwu City, China. Through semi-structured interviews with 27 GPs, six principal themes were identified: acceptance of digital technology, social support networks, integration within the healthcare system, implementation challenges, cultural and contextual adaptation, and recommendations for policy and practice change. These findings align with the study’s objective of exploring the definition, attitudes, barriers, and integration strategies related to digital social prescribing (DSP) for older adults. The Unified Theory of Acceptance and Use of Technology (UTAUT) provides a robust framework for understanding acceptance via performance expectancy, effort expectancy, social influence, and facilitating conditions, complemented by the Health Belief Model (HBM) (21, 33). The results suggest that digital social prescribing (DSP) may serve as an effective intervention to mitigate social isolation, loneliness, and depressive symptoms among older adults in China’s rapidly aging population (3, 6). Nonetheless, this study also highlights substantial barriers that reflect the global state of digital health development, while emphasizing unique challenges within non-Western contexts. In China, these challenges are intensified by pronounced socioeconomic disparities, including the urban–rural divide, and are shaped by distinct cultural norms, such as family dependency structures. Consequently, the effective implementation of digital social prescribing (DSP) necessitates a culturally sensitive approach that adapts technological design, stakeholder engagement, and support mechanisms to address locally pertinent issues, thereby ensuring equitable and sustainable integration into the healthcare system.

The themes emerging from this study both align with and diverge from the international literature, underscoring the critical role of the cultural context. The perception among GPs of digital social prescribing (DSP) as a “transformative tool” facilitating older adults’ connection to online resources aligns with global optimism regarding technology’s role in overcoming mobility limitations (34). This corresponds to the performance expectancy dimension of UTAUT. GPs believed that digital social prescribing (DSP) could “promote the psychological and social wellbeing of older adults by alleviating their sense of isolation” (21, 35). UK and European studies similarly report GPs’ concerns about workload and information governance (25). Our study participants uniquely emphasized the integration of family support within social networks. This emphasis reflects the centrality of filial piety (Xiao) in Confucian societies, contrasting with the more individualistic support models prevalent in Western digital social prescribing (DSP) literature. Moreover, the successful deployment of digital social prescribing (DSP) via platforms such as WeChat in China (36, 37) illustrates a distinctive pathway compared to European models, which frequently rely on bespoke NHS platforms, highlighting the importance of leveraging culturally dominant digital ecosystems to scale the digital social prescribing (DSP).

Digital literacy has emerged as a global barrier, as evidenced by Korean digital social prescribing (DSP) trials (12) and recent evidence-based maps (38). However, the digital divide in China is more pronounced due to stark urban–rural disparities and heterogeneous formal education levels among the older population, presenting a more acute challenge for the government. This contrasts with many Western contexts, where barriers often relate to generational familiarity with technology rather than fundamental infrastructural or educational deficits. Therefore, cultural adaptation must extend beyond superficial language translation to incorporate low-literacy interface design, intergenerational “digital buddy” programs involving family members, and community-based, face-to-face onboarding processes that align with local trust dynamics. Korean rural digital social prescribing (DSP) pilots demonstrated mental health benefits through app usage but revealed persistent disparities in technological proficiency (12). Similarly, Japanese rural social prescribing initiatives have effectively reduced social detachment among isolated older adults (18). European studies indicate that GPs value social prescribing for self-management but express concerns regarding their workload, confidentiality, and access to patients (25). Web-based platforms for chronic condition management have been recognized for respecting patient preferences and psychosocial needs (23). International research highlights the advantages of digital social prescribing (DSP) in terms of accessibility, timeliness, and personalization via remote data sharing and individualized care plans (39). Our findings enrich the UTAUT framework by integrating HBM’s perceived benefits and emphasizing digital social prescribing (DSP) as a catalyst for individuals with weak social networks during urbanization. Notably, GPs in this study seldom endorsed advanced personalization features such as AI-driven companion robots, as proposed in other guidelines (23, 40).

Older adults may prioritize usability, privacy, and relational factors over clinical efficacy and connectivity, as emphasized by the GPs. Patients with limited digital literacy may perceive digital social prescribing (DSP) technologies as intrusive and express concerns regarding autonomy and data privacy (41). They often regard digital mediation as insufficient compared to face-to-face interactions, a nuance potentially underestimated by GPs (42). Family caregivers constitute an underrepresented stakeholder group with distinct perspectives. While GPs view digital social prescribing (DSP) as a mechanism for reducing workload through automated referrals, caregivers may experience increased responsibilities, including technical troubleshooting and monitoring digital engagement (43). Nevertheless, caregivers may appreciate the digital social prescribing (DSP) remote monitoring capabilities more than GPs. Gathering insights from caregivers in rural settings, such as Yiwu, would enhance our understanding of the feasibility of technological integration. Policymakers adopt a macro-level perspective, focusing on funding, reimbursement schemes, data-sharing protocols, and equity considerations (44). These priorities diverge from those of GPs, who emphasize clinical integration and patient-centered care. Policymakers advocate for cross-sectoral coordination between health and social care systems. Barriers identified by GPs may be perceived differently by other stakeholders: accessibility issues by patients, practical workloads by caregivers, and regulatory compliance by policy-makers. European evidence indicates that general practitioners (GPs) recognize the benefits of social prescribing but express concerns about workload and equity (25). GPs favor tools that enhance convenience and respect patient preferences (23). Online DSP address mobility constraints for older adults; however, concerns remain regarding the exclusion of individuals lacking requisite skills (40). Technologies facilitate remote participation in mutual aid groups and telemedicine (11). This study extends the UTAUT by incorporating technology factors and the perceived benefit component of the HBM. GPs regard digital social prescribing (DSP) as a “trigger” for older adults with weak social networks, although discussions of personalization omit advanced AI features, such as companion robots proposed as care solutions (23, 40).

Although cost and digital literacy are recognized barriers, a more comprehensive analysis of the economic implications is warranted. Initial digital social prescribing (DSP) investments encompass technology infrastructure, platform development, training, and ongoing support, which represent significant challenges in resource-constrained settings. A tiered implementation strategy is recommended to facilitate an equitable scale-up. First, phased rollouts should commence in urban pilot districts with higher digital penetration, followed by expansion into rural areas to allow iterative learning and cost amortization. Second, leveraging existing public infrastructure, including community health centers and public Wi-Fi in village halls, may reduce capital expenditures. Third, exploring cross-subsidization models or public-private partnerships, whereby profitable urban deployments subsidize rural implementations, may be viable. Training community health workers and volunteers as “digital navigators” offers a cost-effective approach to bridging literacy gaps, which is applicable to similar initiatives in low-resource contexts (39, 45). Economic evaluations assessing the potential of digital social prescribing (DSP) in reducing downstream healthcare utilization associated with loneliness-related morbidity are essential to justify sustained investment. The GPs’ emphasis on the technological environment reflects these challenges. For example, Japan’s rural digital social prescribing (DSP) pilots demonstrated the technology’s efficacy in alleviating loneliness, contingent on tailored support (18). The benefits of digitization, including accessibility, timeliness, and individualization, identified in other studies, were reaffirmed by the participants (39). Nonetheless, GPs expressed reservations regarding the intervention’s suitability for the oldest-old and individuals with low socioeconomic status (SES) (40).

In the Chinese context, digital social prescribing (DSP) adaptation is influenced by collectivist values, filial piety, and household health production, in contrast to Western models that prioritize individualism and autonomy-focused, primary care-led approaches, such as those prevalent in the UK (41). In Asian countries, including China, Singapore, Japan, and South Korea, digital social prescribing (DSP) implementations often integrate family dynamics and community cohesion, aligning more closely with national aging priorities than with biomedical frameworks (42). Adaptation strategies include customizing content for traditional values, cultural heritage, and digital literacy, and promoting community-based online engagement to bridge urban–rural gaps (43). These culturally attuned modifications may enhance acceptance and equity, distinguishing the Chinese digital social prescribing (DSP) model from its more individualistic international counterparts.

The initial investment for digital social prescribing (DSP), which includes technology infrastructure, platform development, training, and ongoing support, constitutes a significant barrier in resource-limited settings. A tiered implementation approach is recommended to ensure equitable scaling (44). This involves initiating phased rollouts in urban pilot districts with greater digital penetration before extending them to rural areas, thereby enabling iterative learning and cost amortization. Leveraging existing public infrastructure, such as community health centers and public Wi-Fi in village halls, can help to reduce capital costs (46). Additionally, exploring cross-subsidization or public-private partnerships, wherein profitable urban deployments subsidize rural implementations, may be feasible (47, 48). Training community health workers and volunteers as “digital navigators” remains a cost-effective strategy for addressing digital literacy gaps, drawing lessons from comparable initiatives in low-resource environments (39, 45, 49, 50). Economic evaluations quantifying the capacity of digital social prescribing (DSP) to reduce healthcare utilization related to loneliness are critical for advocating sustained funding. GPs’ focus of GPs on the technological environment underscores these global challenges. For instance, Japan’s rural digital social prescribing (DSP) pilots demonstrated the effectiveness of technology in mitigating loneliness, provided that tailored support is available (18). Participants echoed prior findings regarding the benefits of digitization in terms of accessibility, timeliness, and individualization (39). However, concerns persist regarding the intervention’s appropriateness for the oldest and socioeconomically disadvantaged populations (40).

## Conclusion

Our results showed that there was a good attitude toward practitioners who understood the importance of easy-to-use platform applications in promoting social connectivity and alleviating the medical burden on older adults. Digital social prescribing (DSP) has the potential to promote access to services and support for those who cannot leave their homes and those living far away from service locations. GPs revealed that digital social prescribing (DSP) is used as a solution to tackle key problems such as social isolation, loneliness, and poor mental health among older adults. They also identified some barriers related to digital social prescribing (DSP), which reminds us to pay more attention to measures aimed at solving such problems and achieving equal development in the future. The promotion mechanism is explained by considering all the aforementioned promotion factors, including training and policies, based on the technology acceptance model (TAM) and the health belief model (HBM). Digital social prescribing (DSP) accepted by older adults will not only benefit their wellbeing but also empower seniors in this highly technology-dependent society. The development of digital social prescribing (DSP) helps alleviate the social pressure caused by population aging, as its cost-effectiveness is evident in the decrease in hospitalization expenses.

### Policy and practical implications

Our results offer the following actionable recommendations.

1. Policymakers and healthcare administrators should prioritize integrating the Unified Theory of Acceptance and Use of Technology (UTAUT) framework into non-Western healthcare systems to enhance the adoption of digital tools. This involves developing targeted training programs to address barriers such as digital literacy, thereby facilitating the widespread implementation of digital social prescribing (DSP) applications.
2. Based on the hybrid UTAUT-Health Belief Model (UTAUT-HBM), healthcare providers should leverage performance expectancy factors, such as reducing patient loneliness through virtual communities, to foster positive attitudes among general practitioners (GPs) toward digital social prescribing (DSP) applications. Concurrently, efforts to minimize effort expectancy should include user-friendly design enhancements and support resources to overcome usability challenges.
3. To promote digital social prescribing (DSP) in clinical practice, stakeholders should establish guidelines for its application, including pilot programs that incorporate GPs’ (GPs) feedback to refine app functionalities. Globally, governments in Europe and North America, where traditional social prescribing is established, should invest in developing digital versions of these services, drawing on cross-cultural insights from non-Western contexts to encourage international collaboration and knowledge exchange.
4. To translate GPs’ perspectives on digital social prescribing (DSP) into practical solutions, healthcare organizations should conduct iterative consultations with practitioners, leading to the creation of evidence-based protocols that integrate digital social prescribing (DSP) into routine care, ultimately improving patient outcomes and system efficiency.

### Limitations

The design and scope of this study have some inevitable limitations. The sample size of this study was small (*n* = 27). Data were collected using convenience sampling from Yiwu City in Zhejiang Province, which limits the generalizability of the findings. The qualitative, exploratory design does not allow for causal inference or quantification of attitudes, as it is only suitable for a detailed thematic analysis. GPs may be inclined to describe more positive attitudes toward change to conform either with what they think is expected of them as ‘good’ doctors or because they believe such changes are taking place. The sample demographics also showed another limitation. The majority of respondents were male (66.7%) and urban-based (37.0%), with mixed seniority years, possibly not capturing voices from women and countryside GPs, who usually take care of older people living in hard-to-reach communities. There were also no patient or stakeholder views included, which could have provided an overall picture, given that general practitioners’ viewpoints do not necessarily represent those of service users, a similar issue to previous digital social prescribing (DSPs).

### Future research directions

To address the current study’s exclusive focus on GP perspectives and validate the proposed implementation strategies, future research should adopt a multi-stakeholder approach. We outline the following research agenda: First, qualitative comparative studies involving older adult patients and their caregivers are needed to juxtapose their experiences and concerns with GP’s perceptions. Specifically, phenomenological research exploring older patients’ lived experiences with digital health technologies in Yiwu would illuminate whether the “effort expectancy” barriers identified by GPs align with actual patient difficulties or fears. Similarly, focus groups with family caregivers would clarify the practical burdens and benefits of digital social prescribing (DSP) from an informal care perspective. Second, mixed-methods implementation studies should pilot digital social prescribing (DSP) interventions that incorporate feedback loops from all three stakeholder groups (clinicians, patients, and caregivers) during the design phase. These studies should employ participatory action research methodologies, ensuring that patients and caregivers co-design interface features and referral pathways, while policymakers contribute to the scalability assessments. Third, policy analysis research is required to examine the regulatory and funding frameworks necessary for digital social prescribing (DSP) sustainability from policymakers’ perspectives. This should include comparative policy studies between Chinese contexts and other non-Western settings to identify best practices for the cross-sectoral integration of health and social care data systems. Fourth, longitudinal cohort studies tracking digital social prescribing (DSP) outcomes should incorporate multi-informant data collection to simultaneously measure the impact of GP workflow, patient health outcomes, caregiver burden, and cost-effectiveness metrics relevant to health system administrators. Such research would provide a comprehensive evidence base to determine whether the promising clinical perspectives identified in the current study translate into real-world benefits for all stakeholder groups. Finally, dissemination and implementation science research should explore strategies for aligning the divergent priorities of stakeholders, such as reconciling GPs’ focus on clinical efficiency with patients’ desires for human connection and policymakers’ cost constraints through adaptive implementation frameworks tailored to China’s unique urban–rural healthcare contexts.

## Data Availability

The raw data supporting the conclusions of this article will be made available upon reasonable request from the corresponding authors.
