# Supplementary material for: Thematic analysis of how general practitioners perceive digital social prescribing as an intervention aiming at promoting psychosocial health and wellbeing in older adults
Source: Front Public Health. 2026 Mar 25;14:1754026. doi: 10.3389/fpubh.2026.1754026 (PMC13056666; doi:10.3389/fpubh.2026.1754026)
Supplement: Supplementary file 1 [file Table_1.DOCX]

**General Practitioners' Perceptions and Attitudes towards Digital Social Prescribing for Psychosocial Health and Well-being among the Geriatric Population: A Thematic Analysis**

**Interview Guide**

***Section 1: Demographic Questions***

1. **What is your gender?**
2. Male
3. Female
4. Non-binary
5. Prefer not to say
6. **What is your age group?**
7. Under 30
8. 30-39
9. 40-49
10. 50-59
11. 60 or older
12. **How many years have you been practicing as a GP?**
13. Less than 5 years
14. 5-10 years
15. 11-15 years
16. 16-20 years
17. More than 20 years
18. **What is your primary practice setting?**
19. Urban clinic
20. Rural clinic
21. Hospital-based
22. Community health center
23. Other
24. **Approximately what percentage of your patients are aged ≥ 65 years?**
25. Less than 25%
26. 25-49%
27. 50-74%
28. 75% or more

***Section 2: Main Interview Questions***

1. **How would you define digital social prescribing in your own words, particularly in the context of supporting psychosocial health among older adults?**

**Probes:** What comes to mind when you think of digital tools such as apps or online platforms for connecting patients to community activities or virtual support groups? How does it differ from traditional social prescribing? What specific technologies or platforms are considered for older adults? How has your understanding changed with recent technological advancements or patient cases? In your opinion, what role does technology play in addressing psychosocial issues such as social isolation?

1. **To what extent are you aware of digital social prescribing options available for geriatric patients, such as those addressing loneliness, anxiety, and social isolation?**

**Probes:** Can you describe any specific examples that you have encountered? What sources have informed your understanding (e.g., training, colleagues or media)? What experiences, if any, have shaped your familiarity with these tools? How do you stay updated on new digital psychosocial support options? Please elaborate on any gaps in awareness that you have noticed among your peers.

1. **What are your overall attitudes toward using digital social prescribing to improve psychosocial well-being in older adults, such as reducing loneliness or enhancing their quality of life?**

**Probes:** In what ways do you think it could be beneficial for issues such as social isolation or anxiety? How effective do you perceive these digital tools to be compared with in-person options? Can you provide a real-life example of how a digital tool has positively impacted the well-being of an older patient? What role do you see these playing in long-term outcomes, such as reduced anxiety? How might patient demographics influence these benefits?

1. **From your experience, what facilitators or positive factors might encourage the use of digital tools, such as virtual support groups or digital skills programs, for geriatric patients?**

**Probes:** How might these tools help improve accessibility for patients with mobility limitations? What benefits do you see for patient outcomes, such as improved mental health or reduced healthcare visits? How might patient feedback influence your views on these benefits? In what ways could cultural or regional factors in Yiwu enhance their effectiveness? Describe a situation in which a facilitator made a difference.

1. **What barriers do you perceive in implementing digital social prescribing for psychosocial needs in older adults, such as digital access issues or impacts on your workload?**

**Probes:** How might factors such as patients' digital literacy, technology availability, or privacy concerns play a role? Are there any barriers related to the healthcare system or cultural context in Yiwu, China? What difficulties have you noted in older adults' technology adoption, such as interface design? How do workload or reimbursement issues interplay with these factors? Can you provide an example of a practical barrier?

1. **How do you think these barriers can be overcome to make digital social prescribing more feasible?**

**Probes:** What strategies or support (e.g., training for GPs or patient education) would help address issues such as digital divides? Do you have any recommendations for improving digital tools specifically for geriatric patients? What role could collaborations with tech developers play? Have you encountered any successful strategies from your colleagues or training? How might policy changes support these solutions?

1. **How do you envision the integration of digital social prescribing tools into routine care for older patients facing psychosocial challenges?**

**Probes:** What steps would be involved in referring a patient to an app or an online platform? How might this fit into your current workflow, and what workflow changes are needed for seamless referrals? How might you measure success after the implementation? Please provide more information regarding the potential challenges in daily integration.

1. **Based on your views, what improvements or changes would you recommend enhancing the adoption and effectiveness of digital social prescribing in geriatric care?**

**Probes:** Are there any specific features you would like to see in these tools? How can policymakers or developers support GPs in this area? What features, such as user-friendly interfaces for low-literacy users, would you prioritize? How can policy support from health authorities facilitate this process? Which training formats would be most effective for you?
